# Increased Glucose Transport into Neurons Rescues Aβ Toxicity in *Drosophila*

**DOI:** 10.1016/j.cub.2016.09.018

**Published:** 2016-09-26

**Authors:** Teresa Niccoli, Melissa Cabecinha, Anna Tillmann, Fiona Kerr, Chi T. Wong, Dalia Cardenes, Alec J. Vincent, Lucia Bettedi, Li Li, Sebastian Grönke, Jacqueline Dols, Linda Partridge

(Current Biology *26*, 2291–2300; September 12, 2016)

In this article, the neurons shown in Figure 1D were labeled with mtdTomato-3×HA, not GFP as originally stated in the Figure 1D legend and text online and in print. This error, which does not affect the interpretation of our results, has now been corrected in the article online. The authors apologize for the error.

