# Supplementary material for: Increased Glucose Transport into Neurons Rescues Aβ Toxicity in Drosophila
Source: Curr Biol. 2016 Sep 12;26(17):2291–300. doi: 10.1016/j.cub.2016.07.017 (PMC5026704; doi:10.1016/j.cub.2016.07.017)
Supplement: Document S1. Figures S1 and S2 and Supplemental Experimental Procedures [file mmc1.pdf]

**Current Biology, Volume *26***

## Supplemental Information

# Increased Glucose Transport into Neurons Rescues Aβ Toxicity in *Drosophila*

**Teresa Niccoli, Melissa Cabecinha, Anna Tillmann, Fiona Kerr, Chi T. Wong, Dalia Cardenes, Alec J. Vincent, Lucia Bettedi, Li Li, Sebastian Grönke, Jacqueline Dols, and Linda Partridge**

## Supplemental Figures

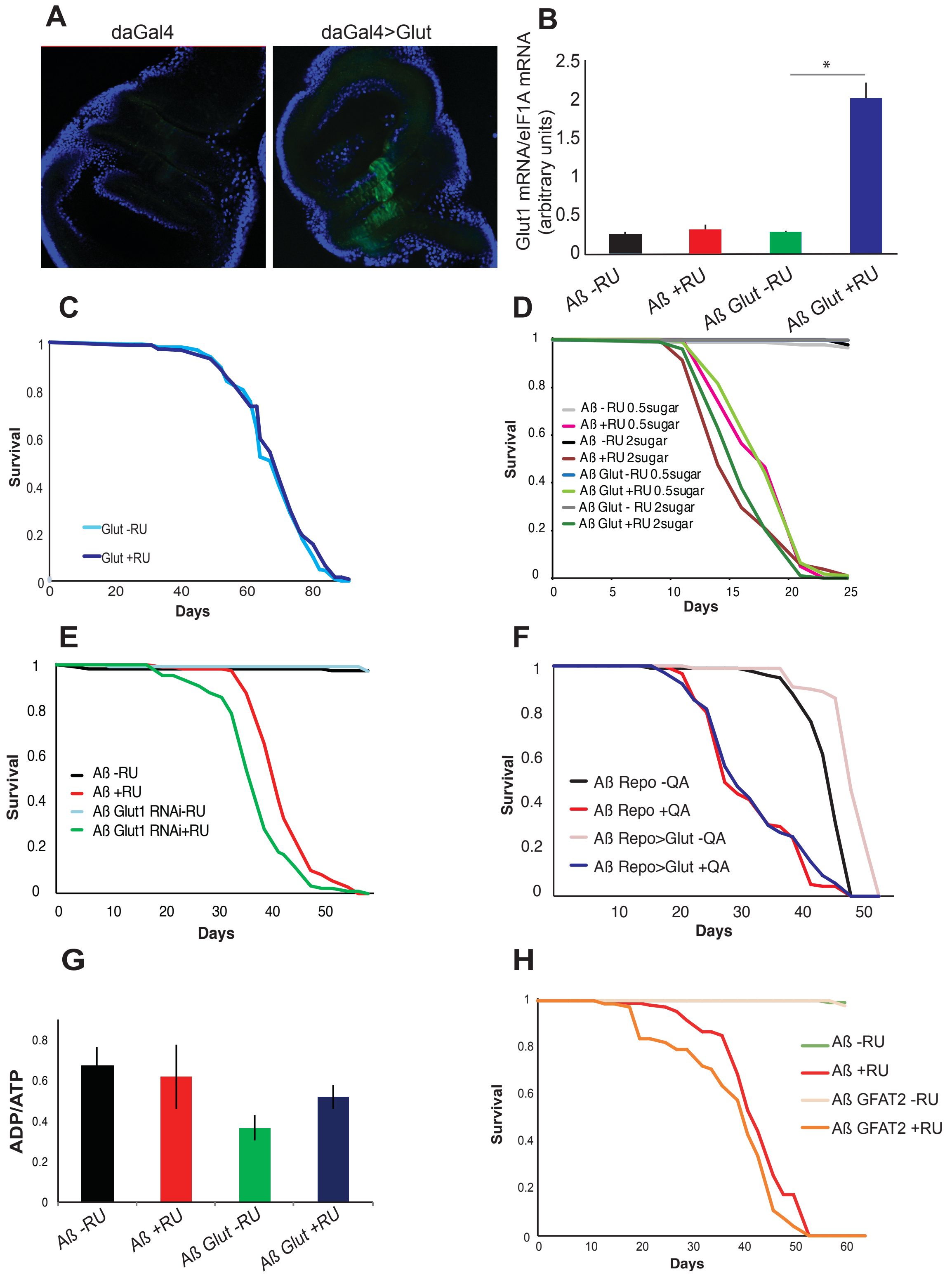


**Supplemental Figure 1, related to Fig 1. A.** Uptake of the fluorescent glucose analogue 2-NBDG (green) in imaginal discs expressing Glut1 and driver alone control (nuclei marked with DAPI in blue). **B**. Glut1 mRNA levels measured by qPCR (relative to eIF1A), showing increase in Glut1 levels in response to RU in the UAS-Glut1 line. Data are presented as means ±SEM (n=3) (* $p \leq 0.01$ by ANOVA) Genotypes: *UAS Aβ; elavGS, UAS Aβ UAS Glut1; elavGS*. **C**. Survival curve of flies expressing Glut1 (+RU) and uninduced control (-RU) in adult neurons.

Genotype: *UAS-Glut1; elavGS.* **D.** Survival curve of flies expressing Aß or Aß Glut1 in adult neurons (+RU) and their uninduced control (-RU), at 29C, on different sucrose concentrations (2=10% and 0.5=2.5%). Sugar concentration differentially modifies the response to RU in Aß Glut1 flies relative to the Aß alone expressing flies ($p<0.05$ by Cox proportional hazards for the interaction between RU, Sugar and genotype). For the individual sugar concentrations comparison on +RU, the lifespans on 0.5x were not statistically significantly different, whereas for 2x sugar, the AßGlut1 lifespan was statistically significantly longer than the Aß alone lifespan ($p\leq0.01$ by Cox proportional hazards analysis). **E.** Survival curves of Aß expressing flies in the presence and absence of Glut1. Aß Glut1RNAi is significantly shorter lived than the Aß alone ($p<0.001$ by log-rank for difference between Aß and Aß Glut1RNAi +RU). Genotypes*: wv; UAS Aβ; elavGS, wv; UAS Aβ; UAS Glut1RNAi/ elavGS.* **F.** Survival curves of flies expressing Glut1 in glia under the control of the repo promoter, while Aß was induced by feeding flies QA. Genotypes*: QUAS Aβ; repoGal4 nSybQF2 tubQS, QUAS Aβ/UAS Glut1; repoGal4 nSybQF2 tubQS* **G.** ADP/ATP ratio in day 19 fly heads expressing Aß or Aß Glut1.There is no statistical difference between the samples. Data are presented as means ±SEM (n=4). Genotypes: *UAS Aβ; elavGS, UAS Aβ/UAS Glut1; elavGS*. **H**. GFAT2 expression shortens Aß lifespan. Survival curves of flies expressing Aß or Aß GFAT2 in neurons induced after eclosion (+RU) and uninduced controls (-RU). Genotypes: *UAS Aβ/ UAS GFAT2; elavGS, UAS Aβ; elavGS*. ($p<0.001$ for difference between Aß +RU and AßGFAT2 +RU by log-rank test)

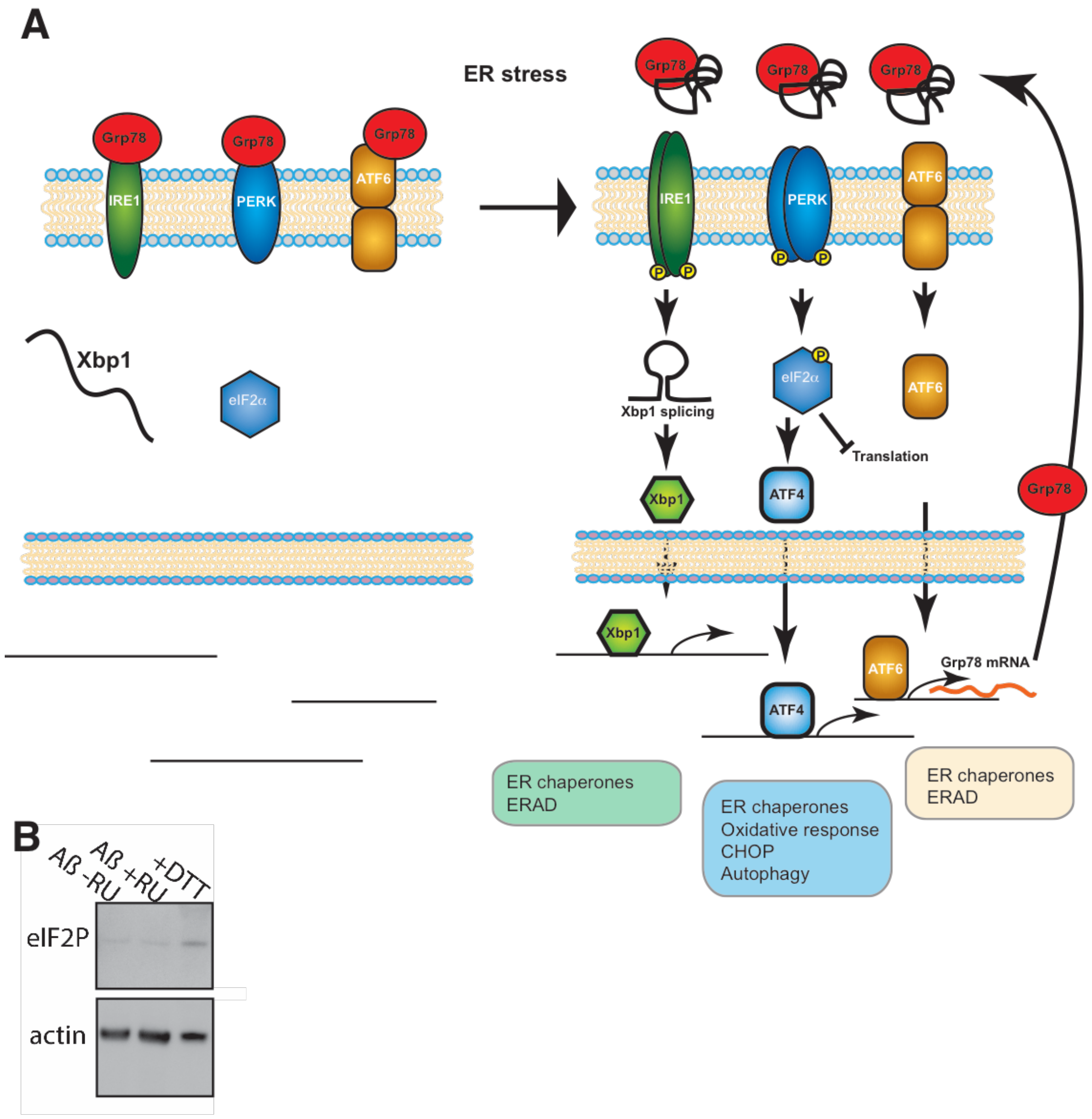


**Supplemental Figure 2, related to Fig 2. A. Schematic representation of the UPR**. The UPR is mediated by 3 trans-membrane signalling components: inositol-requiring enzyme 1 (IRE1), activating transcription factor 6 (ATF6) and protein kinase RNA-like ER kinase (PERK). Under normal physiological conditions, these are bound and inhibited by Grp78/BiP. During ER stress mis-folded proteins titrate away Grp78 thus activating the signalling cascades downstream of IRE1, ATF6 and PERK. This leads to phosphorylation of eIF2a by PERK and global attenuation of protein translation and induction of a set of downstream transcription factors which lead to the production of proteins involved in re-instating ER proteostasis. IRE1 induces Xbp1 splicing, leading to the translation of an active Xbp1transcription factor, ATF6 is cleaved in the Golgi to give a cytosolic ATF6 fragment, which also enters the nucleus to induce transcription, and PERK induces the expression of ATF4. All these induce the expression of chaperones, factors involved in ERAD (ER-associated protein degradation), autophagy, redox and, eventually, apoptosis. **B.**Western blot of head samples from flies expressing Aß (+RU) and their uninduced controls (-RU) as well as heads treated with 5mM DTT in Schneider's medium for 4 hours, to induce the UPR. Genotypes: *UAS Aß; elavGS.*.

## Supplemental Experimental Procedures

### Generation of new transgenic lines

The Glut1 open reading frame from clone LD20062 (DSHB, Iowa) was digested with EcoR1 and Xho1 restriction enzymes and cloned into pUAST. The pUAST-Glut1 construct was used to generate germline transgenics in the w1118 background (Fly Facility, Department of Genetics, University of Cambridge). A number of transgenics were generated and one line carrying UAS-Glut1 (line1) on the second chromosome was used for this study. Another line UAS-Glut1 (line8) was used to confirm the initial phenotype (data not shown).
The GFAT2 open reading frame was amplified from genomic DNA using primers CTACTCCACGGTCACCGACTTG+ CACCATGTGTGGCATATTCGCGT and topo cloned into pENTR/ TOPO entry vector (Invitrogen K2400-20) following the manufacturer's protocol and from this transferred to vector pUASTattB with an LR clonase (Invitrogen 11791-043) according to the manufacturer's protocols. To generate transgenic flies, the vector was integrated into the attP40 landing site using φC31 and attP/attB targeted integration system.
The QUAS-Aß42 Arctic flies were generated by amplifying the Aß42 Arctic sequence from plasmid pMT AßArc (a generous gift from Damian Crowther) with primers CACCATGGCGAGCAAAGTCTC and TTACGCAATCACCACGCCGC and cloned into vector pQUAS. pQUAS-Aß42 Arctic was used to generate germline transgenics in the w1118 background (Fly Facility, Department of Genetics, University of Cambridge). A number of transgenics were generated and one line carrying QUAS-Aß42 Arc (QUAS-Aß) on the second chromosome was used for this study (line D).

### Glucose uptake assay

Glucose uptake was carried out based on [S1]. Briefly, imaginal discs were dissected from L3 larvae and incubated in PBS with 2.5mg/ml 2-NBDG (Molecular Probes N13195) for 20 minutes, then washed twice in ice cold PBS, mounted in Vectashield containing DAPI and imaged immediately on a Zeiss LSM510 inverted confocal microscope.

### ADP/ATP

20 fly heads were homogenised in 30µl of TCA with glass beads in a ribolyser. Samples were spun at 11,500g for 15 minutes at 4ºC. Supernatants were neutralised by diluting 1:10 in 1M Tris HCl pH 8. 50µl of sample was used to measure ADP and ATP with Abcam's ADP/ATP Ratio Assay Kit (ab65313), according to the manufacturer's protocol. Samples were loaded in duplicate, and 4 biological repeats were used for each genotype. Values are expressed as the mean of those replicates +/-SEM, samples were compared with ANOVA.

### Quantitative PCR

Total RNA was extracted from 20-25 fly heads per sample using TRIzol® (GIBCO) according to the manufacturer's instructions. The concentration of total RNA purified for each sample was measured using an Eppendorf biophotometer. One microgram of total RNA was then subjected to DNA digestion using DNase I (Ambion), immediately followed by reverse transcription using the SuperScript® II system (Invitrogen) with oligo(dT) primers. Quantitative PCR was performed using the PRISM 7000 sequence-detection system (Applied Biosystems), SYBR® Green (Molecular Probes), ROX Reference Dye (Invitrogen), and HotStarTaq (Qiagen) by following the manufacturer's instructions. Each sample was analysed in duplicate and values are the mean of three-four independent biological repeats +/- SEM. Primers used were:
Glut1 TTACCGCGGAGCTCTTCTCC +GCCATCCAGTTGACCAGCAC
Aß42 CGATCCTTCTCCTGCTAACC+ CACCATCAAGCCAATAATCG
Grp78 TCTTGTACACACCAACGCAGG+ CAAGGAGCTGGGCACAGTGA
eIF1A ATCAGCTCCGAGGATGACGC + GCCGAGACAGACGTTCCAGA
Spliced Xbp1 CCGAACTGAAGCAGCAACAGC + GTATACCCTGCGGCAGATCC [S2]

**Supplemental References**


S1. Zou, C., Wang, Y., and Shen, Z. (2005). 2-NBDG as a fluorescent indicator for direct glucose uptake measurement. Journal of biochemical and biophysical methods *64*, 207-215.

S2. Maor, G., Rencus-Lazar, S., Filocamo, M., Steller, H., Segal, D., and Horowitz, M. (2013). Unfolded protein response in Gaucher disease: from human to Drosophila. Orphanet J Rare Dis *8*, 140.
