# Supplementary material for: Increased Glucose Transport into Neurons Rescues Aβ Toxicity in Drosophila
Source: Curr Biol. 2016 Sep 12;26(17):2291–300. doi: 10.1016/j.cub.2016.07.017 (PMC5026704; doi:10.1016/j.cub.2016.07.017)
Supplement: Document S2. Article plus Supplemental Information [file mmc2.pdf]

Article

Current Biology

Increased Glucose Transport into Neurons Rescues Aβ Toxicity in *Drosophila*

Highlights

- Overexpression of glucose transporter Glut1 rescues a *Drosophila* Aβ toxicity model
- Glut1 overexpression reduces Grp78 protein levels and induces the UPR
- A Grp78 dominant-negative mutant also rescues Aβ toxicity in *Drosophila*
- Metformin rescues Aβ toxicity and leads to reduced Grp78 expression

Authors

Teresa Niccoli, Melissa Cabecinha, Anna Tillmann, ..., Sebastian Grönke, Jacqueline Dols, Linda Partridge

Correspondence

partridge@age.mpg.de

In Brief

Niccoli et al. show that overexpression of the glucose transporter Glut1 in neurons rescues Aβ toxicity in a *Drosophila* Alzheimer's disease model. This was associated with a reduction in Grp78 levels and induction of the unfolded protein response. This rescue can be mimicked by using metformin, a drug known to induce glucose uptake.

CrossMark

Niccoli et al., 2016, Current Biology *26*, 2291–2300
September 12, 2016 © 2016 The Author(s). Published by Elsevier Ltd.
http://dx.doi.org/10.1016/j.cub.2016.07.017

CellPress

CellPress

Increased Glucose Transport into Neurons Rescues Aβ Toxicity in *Drosophila*

Teresa Niccoli,[1] Melissa Cabecinha,[1] Anna Tillmann,[1] Fiona Kerr,[1] Chi T. Wong,[1] Dalia Cardenes,[1] Alec J. Vincent,[1] Lucia Bettedi,[1] Li Li,[1] Sebastian Grönke,[2] Jacqueline Dols,[2] and Linda Partridge[1,2,*]
[1]Institute of Healthy Ageing, Department of Genetics, Evolution and Environment (GEE), University College London, Darwin Building, Gower Street, London WC1E 6BT, UK
[2]Max Planck Institute for Biology of Ageing, Joseph-Stelzmann-Strasse 9b, 50931 Cologne, Germany
*Correspondence: partridge@age.mpg.de
http://dx.doi.org/10.1016/j.cub.2016.07.017

SUMMARY

Glucose hypometabolism is a prominent feature of the brains of patients with Alzheimer's disease (AD). Disease progression is associated with a reduction in glucose transporters in both neurons and endothelial cells of the blood-brain barrier. However, whether increasing glucose transport into either of these cell types offers therapeutic potential remains unknown. Using an adult-onset *Drosophila* model of Aβ (amyloid beta) toxicity, we show that genetic overexpression of a glucose transporter, specifically in neurons, rescues lifespan, behavioral phenotypes, and neuronal morphology. This amelioration of Aβ toxicity is associated with a reduction in the protein levels of the unfolded protein response (UPR) negative master regulator Grp78 and an increase in the UPR. We further demonstrate that genetic downregulation of Grp78 activity also protects against Aβ toxicity, confirming a causal effect of its alteration on AD-related pathology. Metformin, a drug that stimulates glucose uptake in cells, mimicked these effects, with a concomitant reduction in Grp78 levels and rescue of the shortened lifespan and climbing defects of Aβ-expressing flies. Our findings demonstrate a protective effect of increased neuronal uptake of glucose against Aβ toxicity and highlight Grp78 as a novel therapeutic target for the treatment of AD.

INTRODUCTION

46.8 million people live with dementia worldwide [1], with Alzheimer's disease (AD) being the most common type. Prevalence continues to rise with increasing life expectancy. Currently there are no cures, and there is an urgent need to identify ways of preventing or modifying disease progression. AD is thought to be triggered by the accumulation of extracellular Aβ (amyloid beta) peptides, derived from the misprocessing of amyloid precursor protein (APP) [2], leading to cellular stress, accumulation of toxic intracellular Tau, and eventual neuronal cell death [2]. However, recent evidence suggests that Aβ might also potentially play a protective, antimicrobial role [3].

A prominent feature of AD progression is a substantial reduction in glucose metabolism [4]. This drop precedes the onset of clinical symptoms [4], worsens with disease progression [4], and is a more accurate marker of neuronal atrophy than is Aβ accumulation itself [5]. Patients with type 2 diabetes, who are at higher risk of AD, display increased insulin resistance, which has been linked both to reduced glucose uptake in the brain and to memory impairments [6]. Mouse models of AD also show a decrease in glucose metabolism, suggesting that it may be part of the disease process [7]. However, the exact role of lowered glucose metabolism in disease progression is unknown.

Glucose does not freely cross cell membranes and is, instead, actively shuttled by transporters. In humans, there are 12 glucose transporters, with different expression patterns and affinities. In the brain, Glut1 is expressed mainly in glia and endothelial cells, whereas Glut3 is expressed in neurons [7]. A reduction in expression of a number of glucose transporters has been observed in the brains of mouse AD models [8] and of human patients [7]. The timing of this decrease correlates with increases in Tau phosphorylation and neurofibrillary tangles (NFTs) [7]. In a mouse model of AD pathogenesis, a reduction in neuronal Glut3 expression coincided with a reduction in glucose metabolism [8], while a drop in Glut1 in endothelial cells exacerbated pathology in another mouse AD model [9].

Whether impaired neuronal glucose metabolism plays a causal role in neurodegeneration in AD awaits investigation. The drop in glucose metabolism could contribute to disease progression in several ways. It could lead to a reduction in ATP in neurons, since glucose is the main source of energy. Downregulation of the hexosamine pathway, which relies on glucose for GlcNAc production, would lead to a reduction in Tau GlcNAcylation, which, in turn, could drive up toxic Tau phosphorylation, since the two are negatively correlated [10]. Hypometabolism and glucose deprivation have been shown to induce the unfolded protein response (UPR) [11]; this, too, could drive Tau phosphorylation [11]. Any or all of these mechanisms could contribute to neurodegeneration.

To begin to experimentally test the role of glucose transport and metabolism in AD pathogenesis, we used a model of Aβ toxicity in the fruit fly *Drosophila melanogaster* [12]. *Drosophila* has proved to be an excellent model system in which to study neurodegenerative diseases. The fly has a distinct brain structure with cell types analogous to the human brain, as well as a blood-brain barrier (BBB), and is, therefore, ideal for studying

CrossMark

This is an open access article under the CC BY license (http://creativecommons.org/licenses/by/4.0/).

the neurodegenerative process in a complex tissue. The metabolic coupling between glia and neurons observed in mammalian brains is also conserved in flies [13]. The fly AD model that we used expresses pathogenic Arctic Aβ42 tagged with an endoplasmic reticulum (ER) export signal peptide [14] exclusively in the neurons of the adult fly, thereby removing any confounding developmental effects. These flies have shortened lifespans, behavioral defects, and neurodegeneration [12].

If lowered glucose metabolism in neurons is part of the pathogenic cascade from toxic Aβ, then experimentally increasing glucose metabolism in neurons should ameliorate the pathogenesis in the AD model. Therefore, we assessed the effect of overexpressing a glucose transporter, Glut1, in the neurons of the AD flies. We found that this partially rescued the Aβ phenotypes, without affecting the expression level of the toxic Aβ peptide. Glut1 overexpression led to downregulation of the expression of Grp78 (glucose-regulated protein 78/BiP), the negative master regulator of the UPR. This, in turn, increased the UPR, in association with an improvement in protein homeostasis. Interestingly, feeding the flies the drug metformin, which increases glucose transport, also caused a drop in Grp78 levels and an increase in lifespan, suggesting a possible pharmacological therapeutic avenue.

RESULTS

Drosophila melanogaster has two glucose transporters: Glut1 and Glut3. Glut3 is expressed only in testes, while Glut1 is expressed ubiquitously [15]. In order to increase glucose metabolism, we cloned Glut1 under the control of the UAS (upstream activating sequence) promoter and drove its expression with a constitutive and ubiquitous daGal4 driver. This led to increased uptake of a glucose analog (Figure S1A), demonstrating that increased Glut1 expression can, indeed, increase transport of glucose into cells.

Next, we overexpressed Glut1 in the neurons of adult flies, using an inducible elavGS (elav-GeneSwitch-Gal4) driver and confirmed the overexpression by qPCR in fly heads (Figure S1B). Overexpression of Glut1 had no effect on the lifespan of wild-type flies (Figure S1C), but it increased lifespan in Arctic-Aβ42(Aβ)-expressing flies (Figure 1A) and slowed their decline in climbing ability, a behavioral measure of neuronal health (Figure 1B). Interestingly, the climbing ability of flies expressing Aβ and Glut1 was worse than that of flies expressing Aβ alone at early time points, possibly suggesting that Glut1 expression could impair climbing ability in early life, before its beneficial effect on disease development takes effect (Figure 1B). Sleep pattern, too, is directly controlled by neuronal activity. Flies are diurnal, sleeping mainly at night. Expression of Aβ rendered the flies more arrhythmic, with fewer flies showing a clear change in sleep pattern between day and night, largely as a consequence of a substantial increase in day sleep (Figure 1C). The Aβ flies also spent more total time sleeping than did non-induced controls (Figure 1C). Overexpression of Glut rescued this pattern, with the flies partially recovering their diurnal sleep pattern and spending less total time sleeping (Figure 1C). To confirm that the phenotypic rescue of the Aβ toxicity was, indeed, due to increased glucose uptake and not some other activity of Glut1, we checked whether altering sugar concentration in the food could modulate the Glut1 rescue of lifespan in Aβ-expressing flies (Figure S1D). We found that reducing dietary sugar intake differentially affected the lifespans of flies expressing Aβ alone, relative to the flies expressing Aβ and Glut1, with no rescue of lifespan by Glut 1 at the lowest sugar concentration of 2.5%. As the sugar in the food was reduced, so did the lifespan extension afforded by Glut1. This suggests that the phenotypic rescue is linked to an increased uptake of sugar.

To determine whether Glut1 could rescue neurodegeneration after adult induction of Aβ, we marked a sub-population of neurons with GFP driven by the Q system [16], using nSyb-QF2 > GFP [17], which marks a set of neurons in the central portion of the *Drosophila* brain (Figure 1D) while, at the same time, driving Aβ pan-neuronally with the UAS system. The two misexpression systems are independent of each other, and we could thus monitor the morphology of a sub-population of neurons in the presence of Aβ and of overexpression of Glut1. When Aβ was expressed, a number of filamentous structures clearly visible in the wild-type brain were lost, reflecting the degeneration of axons or dendrites. Strikingly, when Glut1 was overexpressed, the neuronal morphology was completely restored (Figure 1D).

Surprisingly, Glut1 overexpression did not affect Aβ protein or mRNA levels in heads (Figures 1E and 1F), suggesting that Glut1 reduced the toxicity, rather than the total load, of Aβ.

In humans and mouse models of AD, disease progression has been linked to a decrease in glucose transporters [8], and in mouse AD models, a reduction of glucose transporters in endothelial cells exacerbates disease development. Similarly, in our *Drosophila* model, RNAi of Glut1 in neurons (Figure S1E) reduced the lifespan of Aβ-expressing flies, indicating that reduction of glucose import into neurons worsens Aβ toxicity, similar to the mechanism suggested in humans.

In both mammalian and fly brains under normal physiological conditions, glycolysis occurs primarily in glia [13]. Therefore, we assessed whether increasing glucose uptake in glia could also affect pathology. For this, we generated a fly model concomitantly expressing Aβ in neurons and Glut1 in glia. Aβ was driven in neurons by the nSyb-QF2 driver [17], which was induced starting from eclosion, whereas Glut1 was induced by the constitutive glial driver repo-Gal4. Overexpression of Glut1 in glia did not rescue the toxicity of neuronal Aβ, as assessed by lifespan (Figure S1F). Hence, either the Aβ toxicity in neurons was too great for the induction of Glut1 that we achieved to rescue it, or Glut1 in glia cannot rescue Aβ toxicity.

Glucose uptake can influence many cellular processes and could, therefore, rescue Aβ toxicity in several ways. The most obvious candidate is energy metabolism, since glucose is the source of most cellular energy. However, we did not observe an obvious energy deficit in the brains of our Aβ-expressing flies, and the ADP/ATP ratio in brains was unchanged when Glut1 was overexpressed (Figure S1G).

Next, we considered whether Glut1 overexpression reduced Aβ toxicity by acting through the hexosamine biosynthetic pathway, by increasing protein GlcNAcylation. However, upregulation in neurons of GFAT2, the first and rate-limiting enzyme in the hexosamine biosynthetic pathway, shortened the lifespan of the Aβ-expressing flies (Figure S1H), contrary to what would be predicted from this hypothesis.

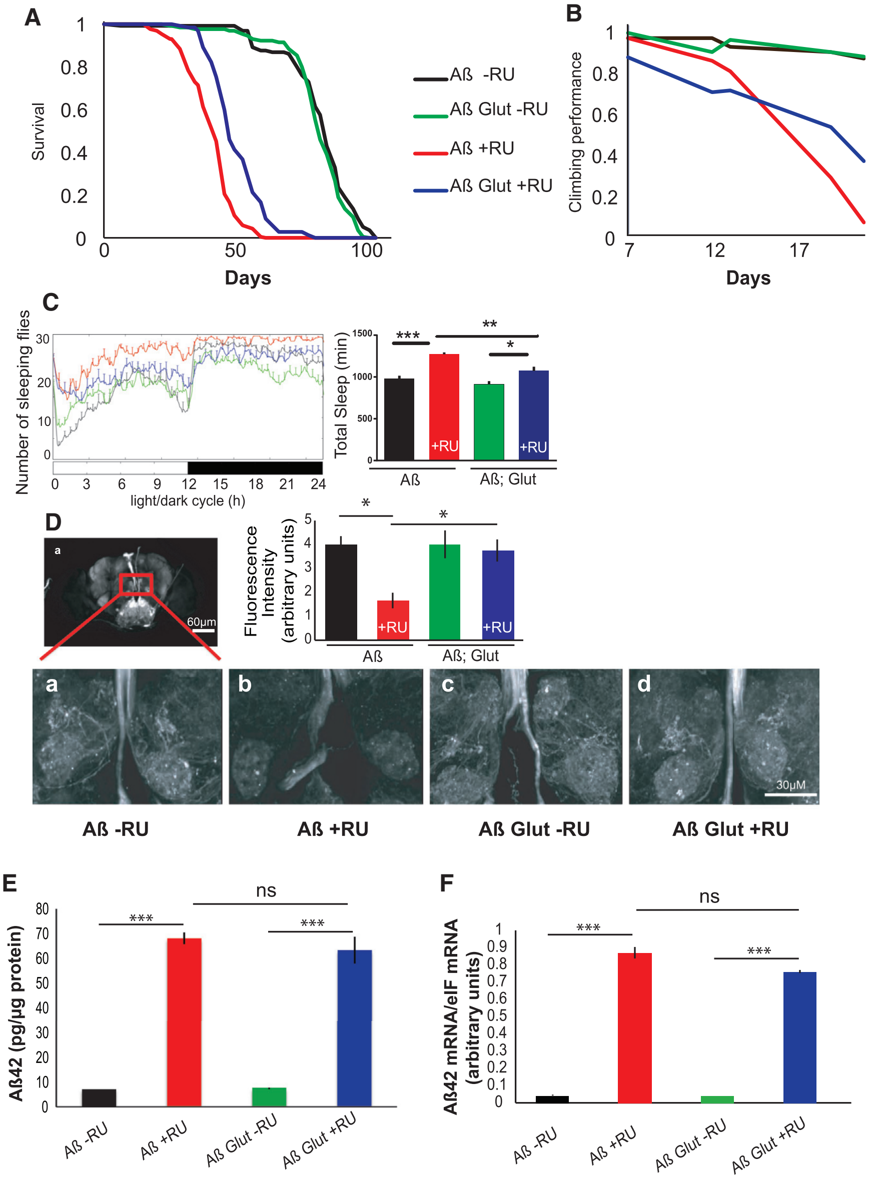

Figure 1. Glut1 Overexpression Rescues Aβ Toxicity

(A) Survival curves of flies expressing Aβ or Aβ Glut1 in adult neurons (+RU) and uninduced controls (-RU). $p < 0.01$, when comparing Aβ +RU and Aβ Glut1 +RU by log-rank test.

(B) Climbing assay performance index of flies of the same genotypes. $p < 1E-10$ when comparing Aβ response to RU relative to Aβ Glut response by ordinal logistics regression.

(C). 24-hr sleep profile of 21-day-old flies expressing Aβ or Aβ Glut1 in neurons (+RU) and uninduced controls (-RU) on day 3 in the LD cycle. At the right, total sleep amount for females of each genotype are shown (plotted as means ± SEM). *$p < 0.01$; **$p < 0.001$; and ***$p < 0.0001$, by two-way ANOVA. Genotypes: *UAS Aβ/UAS Glut1; elavGS*, *UAS Aβ; elavGS*.

(D) Confocal images of brains of 21-day-old control flies (-RU) and flies expressing Aβ or Aβ Glut1 driven by elavGS (+RU). The nSyb-QF2 driver, kept in an inactive state by the tub-QS repressor, drives GFP. Once flies eclosed, they were fed QA, which binds QS to induce expression of GFP, thus labeling a subset of neurons. Fluorescence intensity scores are plotted as means ± SEM. *$p < 0.05$, by ANOVA (n = 3–4). Genotypes: *UAS cd8GFP; nSyb2QF2 tubQS/elavGS*, *UAS cd8GFP/UAS Aβ; elavGS/nSyb2QF2 tubQS*, *UAS cd8GFP/UAS Aβ UAS Glut1; elavGS/nSyb2QF2 tubQS*.

(E) Aβ42 protein levels, measured by ELISA, in the heads of 9-day-old flies expressing Aβ or Aβ Glut1 in neurons (+RU) and uninduced controls (-RU), plotted as means ± SEM (n = 3). ***$p < 0.0001$, by ANOVA; ns, not significant.

(F) Aβ42 mRNA levels (relative to eIF1A) in the heads of similar 14-day-old flies, measured by qPCR, plotted as means ± SEM (n = 4). ***$p < 0.0001$, by ANOVA; ns, not significant. Genotypes: *UAS Aβ; elavGS*, *UAS Aβ/UAS Glut1; elavGS*.

See also Figure S1.

Glucose uptake can affect the UPR [18], which is becoming increasingly recognized as important in neurodegenerative diseases, including AD [19], although whether it plays a protective or detrimental role remains unclear [19]. The UPR signaling cascade is activated in response to ER stress, allowing the cell either to restore protein homeostasis or to enter apoptosis [19], and is mediated by three trans-membrane proteins: pancreatic ER kinase (PERK), inositol-requiring enzyme 1 (IRE1), and activating transcription factor 6 (ATF6). Grp78, also known as BiP, binds and keeps these three proteins in an inactive state. Upon ER stress, Grp78 targets misfolded proteins to act as a chaperone, and thus releases PERK, IRE1, and ATF6 to activate a series of downstream cascades, leading to the phosphorylation of eIF2alpha and reduction of protein translation, activation of downstream transcription factors such as ATF4 and Xbp1, and increases in chaperones such as Grp78 itself (Figure S2A) [19].

The main marker of PERK activation, eIF2alpha phosphorylation, was not affected by either Aβ or Glut1 expression in neurons (Figure 2A); however, we did confirm that the antibody we used was able to detect eIF2alpha phosphorylation in response to a strong UPR inducer (Figure S2B). However, *Grp78* mRNA expression, a marker of ATF6 activation, was increased in flies expressing Aβ (Figure 2B). *Xbp1* splicing, a marker of IRE1 activation, increases in response to Aβ in flies [20]. In our model, we noticed a trend toward an increase in the fluorescence of a GFP reporter for *Xbp1* splicing, but this did not reach significance (Figure 2C). However, when we measured the spliced *Xbp1* isoform by qPCR, it was significantly increased in response to Aβ (Figure 2D), in agreement to what has previously been described [20]. Aβ thus induces the ATF6 and IRE1, but not the PERK, branches of the UPR. Unexpectedly, co-expression of Glut1 increased *Grp78* mRNA (Figure 2B) and *Xbp1* splicing (Figure 2C) even further, suggesting an additional increase of the UPR in

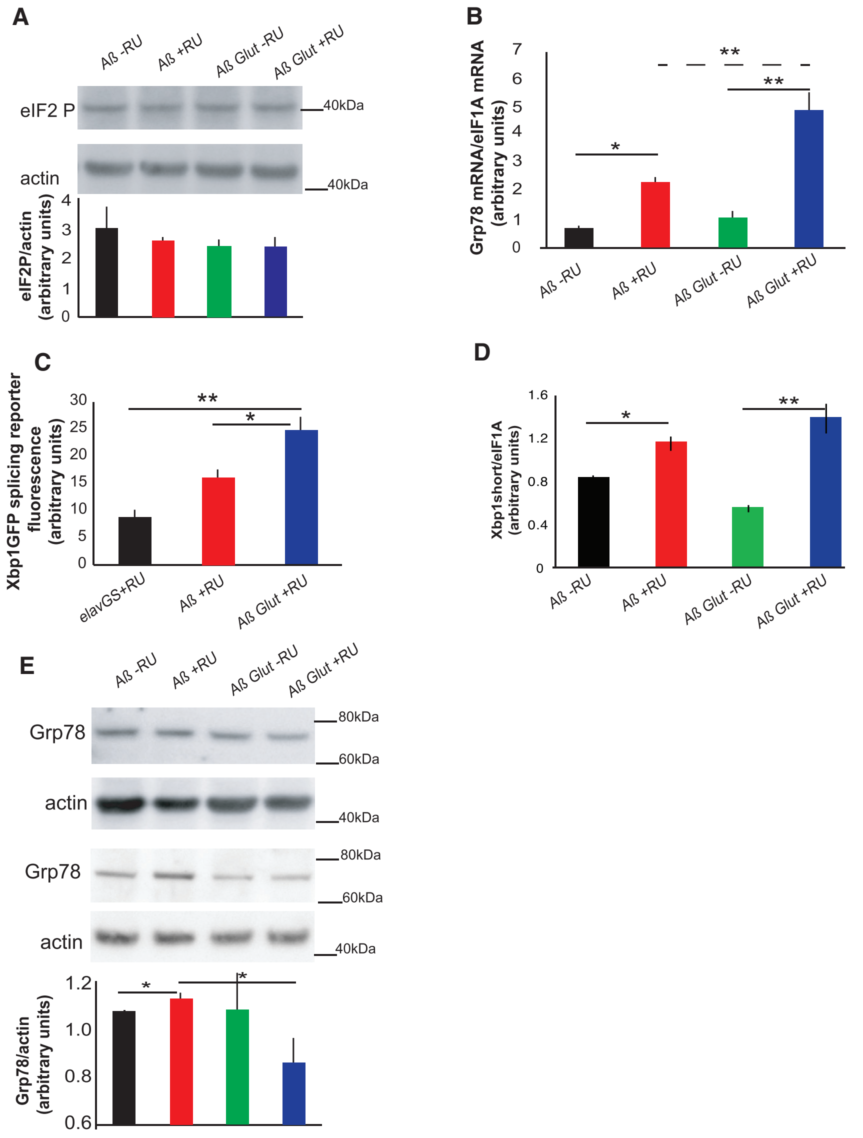

Figure 2. UPR Components Activated in Aβ-Expressing Flies Are Induced Even Further by Glut1 Overexpression

(A) Western blot of eIF2 phosphorylation levels in heads of Aβ- and AβGlut1-expressing flies (+RU) and in controls (-RU), showing no significant difference. Bottom: plotted as means ± SEM (n = 3). Top: a representative gel from the same samples.

(B) Grp78 mRNA levels in heads of 18-day-old flies expressing Aβ or Aβ Glut1 in neurons (+RU) and uninduced controls (-RU), measured by qPCR (relative to eIF1A), plotted as means ± SEM. Genotypes: *UAS Aβ; elavGS*, *UAS Aβ/UAS Glut1; elavGS*.

(C) Quantification of GFP fluorescence in fly brains expressing an Xbp1GFP splicing reporter, plotted as means ± SEM (n = 6–13). Genotypes: *elavGS/UAS-Xbp1GFP*, *UAS Aβ; elavGS/UAS-Xbp1GFP*, *UAS Aβ/UAS Glut1; elavGS/UAS-Xbp1GFP*.

(D) Spliced Xbp1 mRNA levels in heads of 18-day-old flies expressing Aβ or Aβ Glut1 in neurons (+RU) and uninduced controls (-RU), measured by qPCR (relative to eIF1A), plotted as means ± SEM

(E) Western blot of Grp78 in 14-day-old flies of the same genotypes, plotted below as means ± SEM (n = 6–16). The image is a representative gel of the same samples. Genotypes: *UAS Aβ; elavGS*, *UAS Aβ/UAS Glut1; elavGS*.

*p ≤ 0.05; **p ≤ 0.01, by ANOVA. See also Figure S2.

could also increase resistance to tunicamycin. Indeed, Glut1 overexpression protected flies from tunicamycin stress (Figure 3A), accompanied by a block in Grp78 induction (Figure 3B) and a trend toward a further increase in UPR markers upon Glut1 expression (Figures 3C and 3D), similar to what was observed in the Aβ expressing brains. Glut1 could, therefore, protect against UPR stress, attributable to a reduction in Grp78 levels.

response to glucose uptake. Grp78 protein levels are tightly controlled at the level of translation [21], and an increase in the mRNA, therefore, does not necessarily indicate higher protein levels. Therefore, we measured Grp78 protein and found that it also increased in the presence of Aβ, albeit to a smaller extent (Figure 2E). However, surprisingly, overexpression of Glut1 resulted in reduced expression of Grp78 protein (Figure 2E), suggesting that Glut1 and the increased glucose uptake that it produced reduced either Grp78 translation or stability.

Grp78 is broadly considered a negative regulator of the UPR, since it binds and maintains ATF6 and IRE1 in an inactive state. Overexpression of Grp78 can attenuate UPR signaling both in non-neuronal cells [22] and in neurons [19], and its knockdown can lead to increased activation of the UPR upon ER stress [23]. Our results point to a similar mechanism, with a drop in Grp78 protein levels upon Glut1 expression leading to an increase in ATF6 and IRE1 activity. Aβ induced only the IRE1 and ATF6 branches of the UPR, similar to tunicamycin treatment [24]. Therefore, we determined whether Glut1 overexpression

If Glut1 overexpression protects against Aβ-induced UPR stress by reducing Grp78 levels, then a reduction in Grp78 activity should also rescue Aβ toxicity. We tested this by overexpressing a dominant-negative version of Grp78, Grp78K97S, which carries a point mutation affecting the coupling of ATP binding to substrate release [25]. Expression of Grp78K97S in neurons increased both the lifespan and the climbing ability of Aβ-expressing flies (Figures 4A and 4B), suggesting that, indeed, reduced Grp78 activity is causal in the amelioration of Aβ toxicity by Glut1.

AD is characterized by a deregulation of protein homeostasis [26], which could contribute to disease. Therefore, we hypothesized that increased glucose metabolism in neurons allows the UPR to increase even further and, thus, to restore protein homeostasis. To test this idea, we measured insoluble ubiquitinated protein levels in heads and found that Aβ expression led to the accumulation of insoluble ubiquitinated proteins, which was abrogated by Glut1 overexpression (Figure 5), Glut1 overexpression, therefore, allowed neurons to

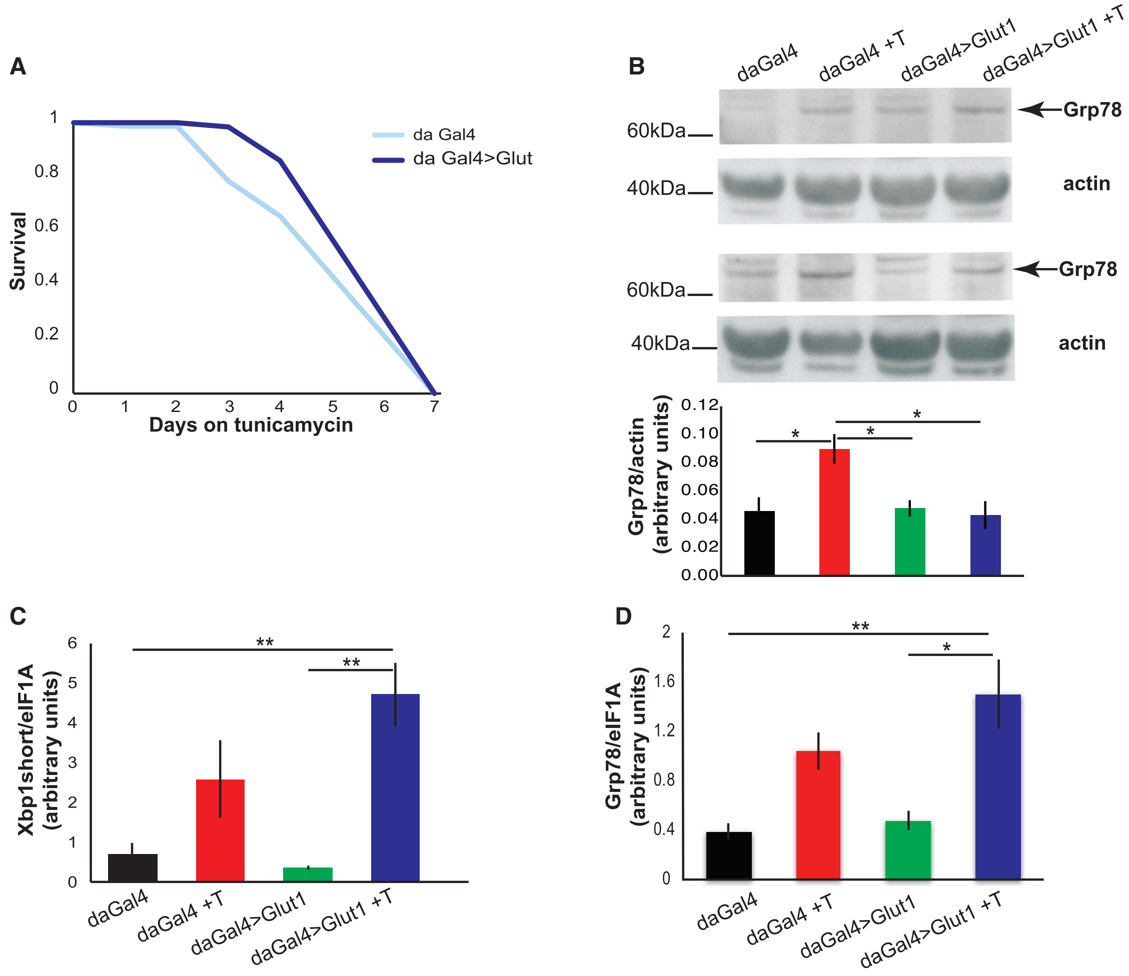

Figure 3. Glut1 Protects Flies from Tunicamycin-Induced ER Stress

(A) Survival of Glut1-overexpressing flies on food containing tunicamycin ($p < 0.05$ for effect of Glut1 relative to driver-alone control by log-rank test).

(B) Western blot of Grp78 in whole flies after 48 hr on tunicamycin, plotted below as means ± SEM (n = 5–8); the image shows representative gels of similar samples. *$p \leq 0.05$ by ANOVA.

(C) qPCR of Xbp1-spliced isoform levels (normalized to eIF1A), in flies treated for 48 hr with tunicamycin, plotted as means ± SEM (n = 3–4). **$p \leq 0.01$ by ANOVA.

(D) qPCR of Grp78 (normalized to eIF1A), in flies treated for 48 hr with tunicamycin, plotted as means ± SEM (n = 3–4). *$p \leq 0.05$; **$p \leq 0.01$, by ANOVA. Genotypes: *daGal4*, *UASGlut1; daGal4*.

re-establish protein homeostasis, presumably via upregulation of the UPR.

Metformin is a drug used to treat type 2 diabetes, and it increases glucose uptake in several tissues [27, 28] by increasing the translocation of glucose transporters to the plasma membrane [27]. Therefore, we treated our Aβ-overexpressing flies with metformin to determine whether we could recapitulate the rescue observed by overexpressing Glut1. Indeed, feeding Aβ-overexpressing flies with a range of metformin concentrations resulted in a significant lifespan extension (Figure 6A) and increase in climbing ability (Figure 6B) without altering Aβ levels (Figure 6C). Interestingly, 80 mM metformin reduced the lifespan of flies that did not express Aβ and was also less effective than lower doses at extending the lifespan of Aβ-expressing flies, but it gave the strongest rescue of climbing ability. These results suggest that this high concentration of metformin shows a beneficial effect on neuronal-related health before 25 days but that continuous exposure to a high dose of metformin leads, at later ages, to systemic toxicity and reduced lifespan. The rescue was dependent on Glut1 expression, since RNAi of Glut1 blocked the lifespan extension from metformin treatment (Figure 6D). Metformin treatment, like Glut1 overexpression, also blocked the increase in Grp78 levels associated with Aβ expression (Figure 6E). Metformin could, therefore, be a potential therapeutic modulator of Aβ pathology by blocking toxic Grp78 induction.

DISCUSSION

Glucose metabolism has been strongly implicated the pathogenesis of AD [5, 7]. Patients display a marked reduction in glucose metabolism in brain areas vulnerable to degeneration, and this precedes the onset of clinical symptoms and mirrors disease progression more closely than does Aβ deposition [4]. Expression of neuronal glucose transporters also drops in AD patients

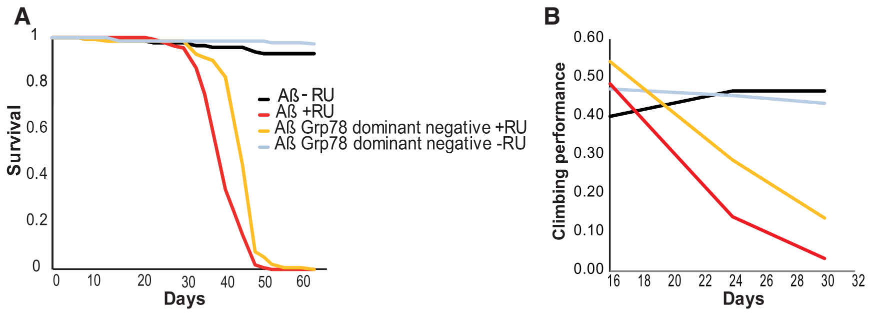

Figure 4. Grp78 Dominant-Negative Version Rescues Aβ Toxicity

(A) Lifespan survival curves of flies expressing Aβ or the Aβ Grp78 dominant-negative version in neurons (+RU) and uninduced controls (-RU). (Aβ +RU and Aβ Grp78 dominant-negative +RU are different. $p < 1E-13$ by log rank).

(B) Climbing assay performance index for the same flies plotted over time ($p < 0.005$ when comparing Aβ response to RU relative to Aβ Grp78.K97S response by ordinal logistics regression). Genotypes: *UAS Aβ; elavGS*, *UAS Aβ/UAS Grp78.K97S; elavGS*.

and in AD mouse models [7]. Recently, it was shown that reduction of glucose transport across the BBB in mouse AD models exacerbates Aβ toxicity [9]. However, whether impaired glucose metabolism in neurons plays a causal role in AD pathogenesis has not been addressed directly.

Our study has shown that experimentally increasing glucose uptake in neurons can protect against Aβ toxicity. In our *Drosophila* AD model, Glut1 overexpression led to a lifespan increase, an amelioration of phenotypes linked to neuronal health—namely, climbing and sleep—and a restoration of normal neuronal morphology. This improvement was associated with a reduction in Grp78 protein levels and an upregulation of the UPR.

In AD patients, the UPR is activated early in disease pathogenesis [11]. Similarly, Aβ expression in the neurons of adult flies led to the induction of the UPR. Grp78 levels were increased, similarly to those of mice models of AD [29], of AD patients early in disease development [30], and in neuronal cells derived from AD patients' induced pluripotent stem cells (iPSCs) [31].

Intriguingly, the rescue of Aβ toxicity by Glut1 was associated with a reduction in Grp78 expression. Grp78 is becoming an increasingly important therapeutic target, especially in cancer biology, where its inhibition increases cells' susceptibility to chemotherapy agents [32]. Its role in neurodegeneration is less well defined. In rat models of Parkinson's disease, activation of Grp78 is protective [33], and its downregulation is detrimental [34]. However, its role in AD models has not been tested. Our studies suggest that Grp78 downregulation can ameliorate Aβ toxicity by allowing upregulation of the UPR. Already, it has been shown in flies that overexpression of Xbp1 can ameliorate Aβ42 toxicity [20], supporting the idea that upregulation of UPR components could be beneficial in AD models. The role of Grp78 has not been tested in other models of neurodegeneration, and a complex picture is emerging regarding the role of downstream UPR effectors, where, depending on the precise experimental conditions, upregulation of the UPR appears to be protective or detrimental [19]. For example, downregulation of PERK is protective in some ALS (amyotrophic lateral sclerosis) or prion disease models, whereas increased Xbp1 can be protective in Parkinson's and Huntington's disease models [19]. This could be because the UPR is a complex pathway with extensive crosstalk between the different signaling cascades, so it would be difficult to predict reliably the outcome of an intervention. Alternatively, a mild upregulation of the UPR could allow the induction of an ER-hormetic response, increasing ER proteostasis to allow a neuron to deal with an increase in misfolded proteins, whereas, in other conditions, a strong induction of the UPR could lead to apoptosis, and, therefore, blocking this response could also increase neuronal survival [19].

How glucose regulates Grp78 expression is unclear. It is well established that glucose starvation induces expression of Grp78 [35], and, in one study, increased glucose reduced Grp78 expression in cultured neurons [36]. However, little is known about how physiological changes in glucose metabolism modulate Grp78, and it will be important to identify the mechanisms at work.

Our study suggests a model where Aβ accumulation in the brain induces the UPR, and increased glucose uptake in neurons blocks the negative feedback loop linked to Grp78 upregulation, resulting in even further increased UPR, which allows neurons to clear insoluble ubiquitinated proteins and restore protein homeostasis, resulting in the rescue of neurodegeneration and increased lifespan. This mechanism of action could be relevant to the benefits observed by administration of insulin nasal spray in AD patients [37]. Insulin is thought to act via PI3K/MAPK (phosphatidylinositol 3-kinase/mitogen-activated protein kinase) to influence Aβ trafficking and decrease Tau phosphorylation by inhibiting Gsk3 [38]. However, insulin can upregulate glucose transporters in neurons [39] and increase glucose metabolism [39]. It would be interesting to determine whether insulin administration also upregulates the UPR.

Glucose metabolism has also been implicated in the increased risk of AD associated with type 2 diabetes. The link is well established, but the mechanism is less so [40]; possibilities include increased vascular risk factors associated with metabolic syndrome in type 2 diabetes, as well as hyperglycemia-linked dysregulation of cellular signaling pathways, leading to advanced glycation products and increased reactive oxygen species. Also, the rise in brain insulin resistance, which could, in part, lead to increased production of Aβ [40], could also result in decreased glucose transporters and glucose metabolism within neurons, leading to an upregulation of Grp78.

Metformin, a type 2 diabetes therapy, is a drug that has been shown to increase glucose uptake in cells. Interestingly, treatment with a metformin dose known not to affect lifespan in wild-type *Drosophila* [41] increased the lifespan of Aβ-expressing flies. In accordance with previous studies [42], we also found that metformin decreased expression of Grp78, suggesting that the rescue of Aβ toxicity could be due to increased UPR. Metformin's effect in AD is controversial, with some studies reporting patient benefits [43] but others finding that it worsened cognitive performance [44], possibly related to metformin's ability to

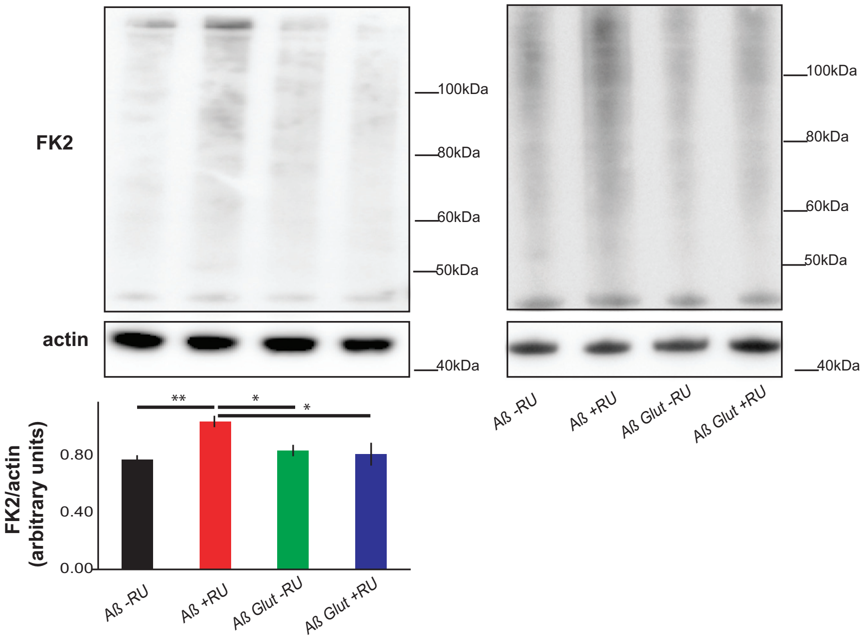

Figure 5. Glut1 Reduces Accumulation of Insoluble Ubiquitinated Proteins

Western blots of SDS-soluble protein fraction from the heads of day-17 flies expressing Aβ or Aβ Glut1 in neurons (+RU) and uninduced controls (-RU), probed for ubiquitinated proteins (FK2) and for actin, plotted below as means ± SEM (n = 4). The two blots shown are from different experiments. *$p < 0.05$; **$p < 0.01$, by ANOVA. Genotypes: *UAS Aβ; elavGS*, *UAS Aβ/UAS Glut1; elavGS*.

increase APP levels and processing to increase Aβ production [45]. Our study points to a novel and unexplored role of metformin as a modulator of the UPR in neurodegeneration downstream of Aβ accumulation, which could provide a useful therapeutic avenue in a clinical context where AD patients present quite late in disease development and have already accumulated Aβ peptide in their brain.

EXPERIMENTAL PROCEDURES

Fly Husbandry and Stocks

All flies were reared at 25°C on a 12-hr:12-hr light:dark (LD) cycle at constant humidity and on standard sugar-yeast-agar (SYA) medium (agar, 15 g/l; sugar, 50 g/l; autolyzed yeast, 100 g/l; nipagin, 100 g/l; and propionic acid, 2 ml/l). Adult-onset, neuron-specific expression of UAS constructs was achieved as described elsewhere [12]. Briefly, 24–48 hr after eclosion, female flies carrying a heterozygous copy of elavGS and at least one UAS construct were fed SYA medium supplemented with 200 μM mifepristone (RU486) to induce transgene expression. For induction with quinic acid (QA), flies were put on food containing 7.5 g of QA per liter. Metformin was added to the food at the stated concentrations. ElavGS was derived from the original elavGS 301.2 line [46] and obtained as a generous gift from Dr. H. Tricoire (CNRS); the UAS-Aβ42Arc (UAS Aβ) stock was a gift from Dr. D. Crowther (University of Cambridge). The nSyb-QF2 stock was a gift from Dr. C. Potter (Johns Hopkins School of Medicine) [17]. W1118, tubulin-QS (tub-QS), UAS-Grp78.K97S, UAS-Xbp1GFP, daughterless-Gal4 (daGal4), Glut1 RNAi line (TRiP.HMS02152) and Repo-Gal4 were obtained from the Bloomington Drosophila Stock Center.

All transgenes were backcrossed into the w1118 background to ensure a homogeneous genetic background between transgenic lines. All experiments were carried out on mated females, unless otherwise stated.

Lifespan Analysis

Flies were raised at standard density in 200-ml bottles. After eclosion, flies were allowed to mate for 24–48 hr. At least 110–150 females of the appropriate genotype were split into groups of 15 and housed in vials containing SYA medium with or without drugs. Deaths were scored, and flies tipped onto fresh food three times a week. Data are presented as cumulative survival curves, and survival rates were compared using log-rank tests or Cox proportional hazards performed in JMP (version 9.0) software (SAS Institute). All lifespans were performed at 25°C unless otherwise stated.

Climbing Assay

The climbing assay in Figure 1 was performed as previously described [47]. Briefly, 15 flies were placed in a 25-cm pipette, tapped to the bottom, and allowed to climb for 45 s. The number of flies in the top 5 cm, center, and bottom 3 cm was scored. A performance index was calculated for each time point and plotted. Statistical analysis was performed in R using ordinal logistics regression, using the individual heights for each fly as data points. For the climbing assay in Figures 4 and 6, the assay was performed with the following modifications: 45 flies were housed in a glass-walled chamber 25 cm tall, and flies were tapped to the bottom as described earlier and allowed to climb for 20 s before scoring. The analysis was the same as described earlier.

Western Blotting

Protein samples were prepared by homogenizing in 2× SDS Laemmli sample (4% SDS, 20% glycerol, 120 mM Tris-HCl [pH 6.8], 200 mM DTT with bromophenol blue) and boiled at 95°C for 5 min. Samples were separated on pre-cast 4%–12% Invitrogen Bis-Tris gels (NP0322) and blotted onto PVDF (polyvinylidene fluoride) or nitrocellulose membrane (for Grp78) in Tris-glycine buffer supplemented with 10% ethanol. Membranes were blocked in 5% milk and 1% BSA in TBS-T (Tris-buffered saline with 0.05% Tween-20) for 1 hr at room temperature (RT) and then incubated with primary antibodies in block. Ubiquitin westerns were blocked in 1% BSA. Primary antibody dilutions used were as follows: anti-Grp78, 1:1,000 (Novus Biologicals, NBP1-06274); anti-actin, 1:10,000 (Abcam, ab1801); anti-Ubiquitin, 1:1,000 (Millipore, FK2); and anti-eIF2A-phospho, 1:1,000 (Cell Signaling, 3597). Secondaries used were anti-rabbit and anti-mouse (Abcam, ab6789 and ab6721) at 1:10,000 dilutions for 1 hr at RT. Bands were visualized with Luminata Forte (Millipore) and imaged with ImageQuant LAS4000 (GE Healthcare Life Sciences). Quantification was carried out with ImageQuant software or ImageJ.

Preparation of Detergent-Soluble Fractions for Insoluble Ubiquitinated Protein Gels

The method was adapted from [48]. Briefly, 10–20 heads were extracted in 75 μl Triton X extraction buffer and spun at 13,000 × *g* for 10 min at 4°C, and the supernatant was collected as the Triton X soluble fraction. The pellet was re-suspended in 50 μl SDS extraction buffer and spun again, and the supernatant was collected as the SDS soluble fraction. Samples were stored at −80°C and run as described earlier.

qPCR

Total RNA was extracted from heads or whole flies (for Figure 3) and converted to cDNA (see Supplemental Experimental Procedures for details). qPCR was performed using the PRISM 7000 sequence detection system (Applied Biosystems). Each sample was analyzed in duplicate, and values are the mean of three or four independent biological repeats ±SEM.

Quantification of Aβ42

Five fly heads were homogenized in 50 ml GnHCl extraction buffer (5 M guanidinium HCl, 50 mM HEPES [pH 7.3], 1:10 dilution of protease inhibitor cocktail

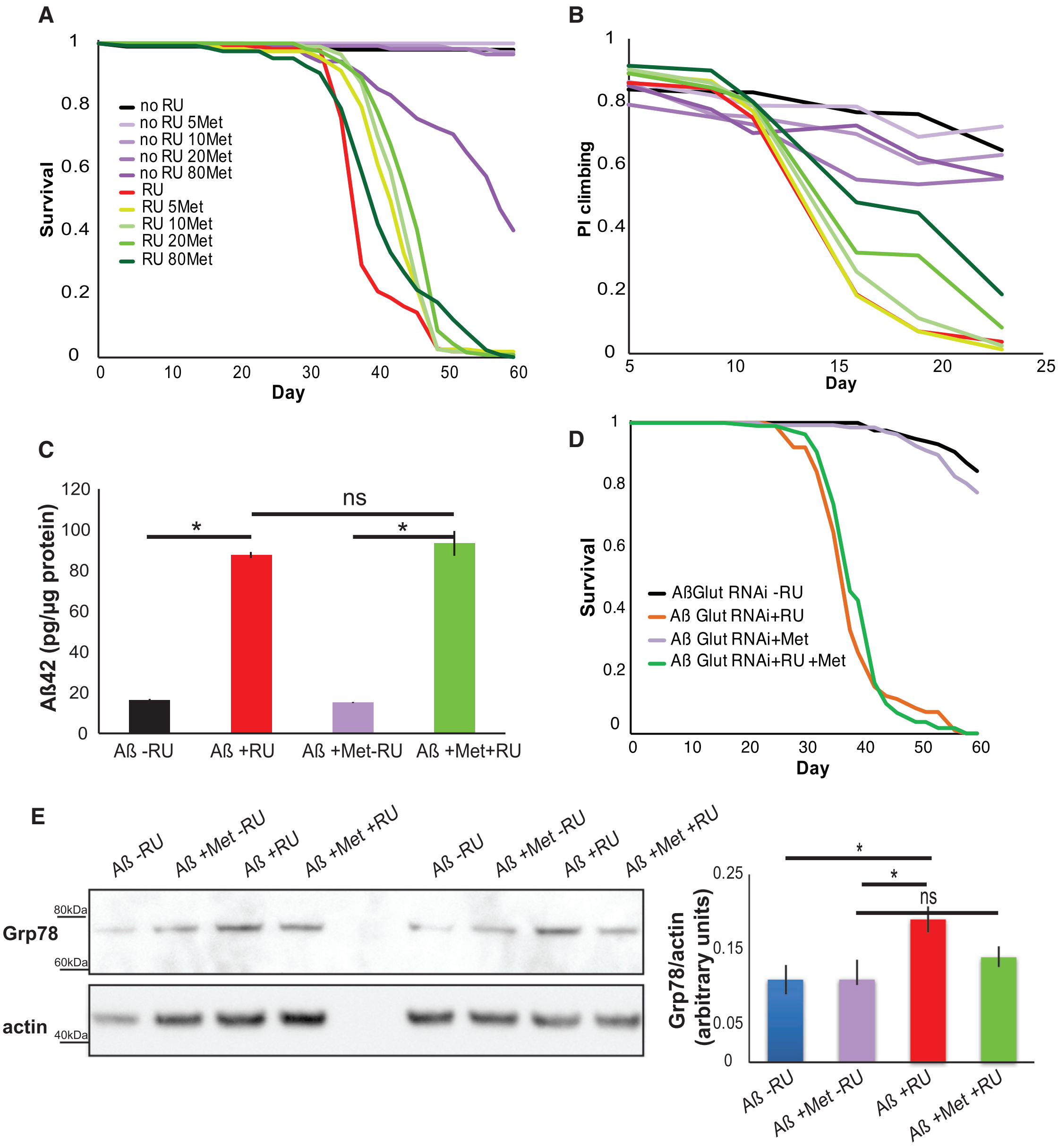

Figure 6. Metformin Extends the Lifespan of Aβ-Expressing Flies

(A) Lifespan survival curves of flies expressing Aβ (+RU) and controls (-RU) in the presence of different metformin (Met) concentrations (all the metformin-treated +RU flies were significantly longer lived than the +RU-alone control: $p < E\text{-}4$ for 5 mM, $p < E\text{-}6$ for 10 mM, $p < E\text{-}8$ for 20 mM, and $p < 0.01$ for 80 mM by log-rank test).

(B) Climbing assay performance index (PI) for the same flies ($p < 1E\text{-}5$ when comparing the +RU control relative to the +RU flies treated with 20 mM or 80 mM metformin by ordinal logistics regression).

(C) Aβ42 protein levels, measured by ELISA, in the heads of 15-day-old flies expressing Aβ (+RU) and controls (-RU) in the presence or absence of 10 mM metformin, plotted as means ± SEM (n = 3). *$p < 0.0001$, by ANOVA; ns, not significant.

(D) Lifespan survival curves of flies expressing Aβ Glut1 RNAi (+RU) and controls (-RU) in the presence or absence of 10 mM metformin (no difference between +RU-treated flies by log-rank test). Genotype: *wv; UAS Aβ; elavGS/Glut1RNAi*. (C).

(E) Western blots for Grp78 and actin control in the heads of flies treated with 10 mM meformin, plotted as means ± SEM (n = 4). *$p < 0.01$, by ANOVA; ns, not significant.

Genotype for (A)–(C) and (E): *UAS Aβ; elavGS*.

[Sigma, P8340], and 5 mM EDTA) and centrifuged at 21,000 × *g* for 5 min at 4°C, and cleared supernatant was retained as the total fly Aβ42 sample. Aβ42 was measured with an ELISA kit (Millipore, EZHS42), according to the manufacturer's instructions, and protein was measured with a Bradford assay (Bio-Rad protein assay reagent), and the amount of Aβ42 in each sample was expressed as a ratio of the total protein content (picograms per microgram of total protein). Data are expressed as the mean ± SEM obtained from three biological repeats for each genotype.

Microscopy

Brains were dissected in 4% paraformaldehyde in PBS with Tween 20 (PBST), incubated for 20 min, rinsed in PBST, mounted in Vectashield with DAPI, and imaged on a Zeiss LSM510 inverted confocal microscope. Images were taken on the 20× or 40× objective as stacks and are shown as maximum intensity projections of the complete stack. The same size stacks were taken for experimental and control samples. All images for one experiment were taken at the same settings. For Xbp1 fluorescence (in Figure 2C), total fluorescence intensity of a given area of the brain was measured with ImageJ. Values shown are the averages for 6–13 brains ± SEM. Samples were compared by ANOVA. For neuronal GFP, images were blind scored and given a score from 1 to 5 based on fluorescent intensity; average scores are presented with their SEM (n = 3–5).

Analysis of Activity and Sleep

Individual, 21-day-old, mated female flies were placed in glass tubes (65 mm × 5 mm) containing standard 1× SYA, and activity was recorded using the DAM System (*Drosophila* Activity Monitoring System; TriKinetics) as described previously [49]. Flies were entrained to a 12-hr:12-hr light:dark (LD) cycle at 25°C and 65% humidity 24–36 hr before recording. 5 days of the 12:12 hr LD cycle were recorded, followed by 5 days of a 12-hr:12-hr dark:dark (DD) cycle. Analysis of locomotor activity was performed using the fly toolbox and MATLAB software (MathWorks), as described previously [50]. Sleep was defined as a bout of inactivity lasting 5 min or more, and sleep analysis was performed with pySOLO [51]. All behavioral data (activity and sleep duration) are represented by mean values with their SEM.

Tunicamycin Stress Assay

Flies were tipped into vials containing 1% agar, 1.5% sucrose, and 10 mg/l of tunicamycin. Dead flies were scored at regular intervals, and lifespan curves were compared with log-rank test.

SUPPLEMENTAL INFORMATION

Supplemental Information includes two figures and Supplemental Experimental Procedures and can be found with this article online at http://dx.doi.org/10.1016/j.cub.2016.07.017.

AUTHOR CONTRIBUTIONS

Conceptualization, T.N. and L.P.; Methodology, T.N., A.T., F.K., and L.P.; Investigation, T.N., M.C., A.T., F.K., L.L., C.T.W., D.C., L.B., and A.J.V.; Writing – Original Draft, T.N.; Writing – Review & Editing, T.N. and L.P.; Funding Acquisition, L.P.; Resources, S.G. and J.D.; Supervision, T.N. and L.P.

ACKNOWLEDGMENTS

We thank Nazif Alic for help with statistical analysis and Nathan Woodling for comments on the manuscript. We also thank the neuro group for helpful discussion and Alexander Hassabis for help with scoring. This work was supported by the Wellcome Trust (grant number WT098565/Z/12/Z to L.P.), the ARUK (grant number ART/PG2009/4 to L.P.), and the Max Planck Society (to L.P.).

Received: November 29, 2015
Revised: June 9, 2016
Accepted: July 11, 2016
Published: August 11, 2016

REFERENCES

1. Prince, M., Wimo, A., Guerchet, M., Ali, G.-C., Wu, Y.-T., and Prina, M.; Alzheimer's Disease International (2015). World Alzheimer Report 2015: The Global Impact of Dementia: An Analysis of Prevalence, Incidence, Cost and Trends. http://www.alz.co.uk/research/WorldAlzheimerReport2015.pdf.
2. Hardy, J.A., and Higgins, G.A. (1992). Alzheimer's disease: the amyloid cascade hypothesis. Science *256*, 184–185.
3. Kumar, D.K., Choi, S.H., Washicosky, K.J., Eimer, W.A., Tucker, S., Ghofrani, J., Lefkowitz, A., McColl, G., Goldstein, L.E., Tanzi, R.E., and Moir, R.D. (2016). Amyloid-β peptide protects against microbial infection in mouse and worm models of Alzheimer's disease. Sci. Transl. Med. *8*, 340ra72.
4. Dukart, J., Kherif, F., Mueller, K., Adaszewski, S., Schroeter, M.L., Frackowiak, R.S., and Draganski, B.; Alzheimer's Disease Neuroimaging Initiative (2013). Generative FDG-PET and MRI model of aging and disease progression in Alzheimer's disease. PLoS Comput. Biol. *9*, e1002987.
5. La Joie, R., Perrotin, A., Barré, L., Hommet, C., Mézenge, F., Ibazizene, M., Camus, V., Abbas, A., Landeau, B., Guilloteau, D., et al. (2012). Region-specific hierarchy between atrophy, hypometabolism, and β-amyloid (Aβ) load in Alzheimer's disease dementia. J. Neurosci. *32*, 16265–16273.
6. Willette, A.A., Bendlin, B.B., Starks, E.J., Birdsill, A.C., Johnson, S.C., Christian, B.T., Okonkwo, O.C., La Rue, A., Hermann, B.P., Koscik, R.L., et al. (2015). Association of insulin resistance with cerebral glucose uptake in late middle-aged adults at risk for Alzheimer disease. JAMA Neurology *72*, 1013–1020.
7. Shah, K., Desilva, S., and Abbruscato, T. (2012). The role of glucose transporters in brain disease: diabetes and Alzheimer's disease. Int. J. Mol. Sci. *13*, 12629–12655.
8. Ding, F., Yao, J., Rettberg, J.R., Chen, S., and Brinton, R.D. (2013). Early decline in glucose transport and metabolism precedes shift to ketogenic system in female aging and Alzheimer's mouse brain: implication for bioenergetic intervention. PLoS ONE *8*, e79977.
9. Winkler, E.A., Nishida, Y., Sagare, A.P., Rege, S.V., Bell, R.D., Perlmutter, D., Sengillo, J.D., Hillman, S., Kong, P., Nelson, A.R., et al. (2015). GLUT1 reductions exacerbate Alzheimer's disease vasculo-neuronal dysfunction and degeneration. Nat. Neurosci. *18*, 521–530.
10. Liu, F., Shi, J., Tanimukai, H., Gu, J., Gu, J., Grundke-Iqbal, I., Iqbal, K., and Gong, C.X. (2009). Reduced O-GlcNAcylation links lower brain glucose metabolism and tau pathology in Alzheimer's disease. Brain *132*, 1820–1832.
11. van der Harg, J.M., Nölle, A., Zwart, R., Boerema, A.S., van Haastert, E.S., Strijkstra, A.M., Hoozemans, J.J., and Scheper, W. (2014). The unfolded protein response mediates reversible tau phosphorylation induced by metabolic stress. Cell Death Dis. *5*, e1393.
12. Sofola, O., Kerr, F., Rogers, I., Killick, R., Augustin, H., Gandy, C., Allen, M.J., Hardy, J., Lovestone, S., and Partridge, L. (2010). Inhibition of GSK-3 ameliorates Abeta pathology in an adult-onset *Drosophila* model of Alzheimer's disease. PLoS Genet. *6*, e1001087.
13. Volkenhoff, A., Weiler, A., Letzel, M., Stehling, M., Klämbt, C., and Schirmeier, S. (2015). Glial glycolysis is essential for neuronal survival in *Drosophila*. Cell Metab. *22*, 437–447.
14. Crowther, D.C., Kinghorn, K.J., Miranda, E., Page, R., Curry, J.A., Duthie, F.A., Gubb, D.C., and Lomas, D.A. (2005). Intraneuronal Abeta, non-amyloid aggregates and neurodegeneration in a *Drosophila* model of Alzheimer's disease. Neuroscience *132*, 123–135.
15. Chintapalli, V.R., Wang, J., and Dow, J.A. (2007). Using FlyAtlas to identify better *Drosophila melanogaster* models of human disease. Nat. Genet. *39*, 715–720.
16. Potter, C.J., and Luo, L. (2011). Using the Q system in *Drosophila melanogaster*. Nat. Protoc. *6*, 1105–1120.
17. Riabinina, O., Luginbuhl, D., Marr, E., Liu, S., Wu, M.N., Luo, L., and Potter, C.J. (2015). Improved and expanded Q-system reagents for genetic manipulations. Nat. Methods *12*, 219–222.
18. Kaufman, R.J. (2002). Orchestrating the unfolded protein response in health and disease. J. Clin. Invest. *110*, 1389–1398.
19. Hetz, C., and Mollereau, B. (2014). Disturbance of endoplasmic reticulum proteostasis in neurodegenerative diseases. Nat. Rev. Neurosci. *15*, 233–249.
20. Casas-Tinto, S., Zhang, Y., Sanchez-Garcia, J., Gomez-Velazquez, M., Rincon-Limas, D.E., and Fernandez-Funez, P. (2011). The ER stress factor

XBP1s prevents amyloid-beta neurotoxicity. Hum. Mol. Genet. *20*, 2144–2160.

21. Gülow, K., Bienert, D., and Haas, I.G. (2002). BiP is feed-back regulated by control of protein translation efficiency. J. Cell Sci. *115*, 2443–2452.
22. Bertolotti, A., Zhang, Y., Hendershot, L.M., Harding, H.P., and Ron, D. (2000). Dynamic interaction of BiP and ER stress transducers in the unfolded-protein response. Nat. Cell Biol. *2*, 326–332.
23. Li, J., Ni, M., Lee, B., Barron, E., Hinton, D.R., and Lee, A.S. (2008). The unfolded protein response regulator GRP78/BiP is required for endoplasmic reticulum integrity and stress-induced autophagy in mammalian cells. Cell Death Differ. *15*, 1460–1471.
24. Shinjo, S., Mizotani, Y., Tashiro, E., and Imoto, M. (2013). Comparative analysis of the expression patterns of UPR-target genes caused by UPR-inducing compounds. Biosci. Biotechnol. Biochem. *77*, 729–735.
25. Elefant, F., and Palter, K.B. (1999). Tissue-specific expression of dominant negative mutant *Drosophila* HSC70 causes developmental defects and lethality. Mol. Biol. Cell *10*, 2101–2117.
26. Borchelt, D.R., Xu, G., Notterpek, L., and Lewis, J. (2014). Proteostasis and secondary proteinopathy in Alzheimer's disease. J. Alzheimers Dis. Parkinsonism *4*, 3.
27. Hundal, H.S., Ramlal, T., Reyes, R., Leiter, L.A., and Klip, A. (1992). Cellular mechanism of metformin action involves glucose transporter translocation from an intracellular pool to the plasma membrane in L6 muscle cells. Endocrinology *131*, 1165–1173.
28. Cooper, A.C., Fleming, I.N., Phyu, S.M., and Smith, T.A. (2015). Changes in [18F]Fluoro-2-deoxy-D-glucose incorporation induced by doxorubicin and anti-HER antibodies by breast cancer cells modulated by co-treatment with metformin and its effects on intracellular signalling. J. Cancer Res. Clin. Oncol. *141*, 1523–1532.
29. Soejima, N., Ohyagi, Y., Nakamura, N., Himeno, E., Iinuma, K.M., Sakae, N., Yamasaki, R., Tabira, T., Murakami, K., Irie, K., et al. (2013). Intracellular accumulation of toxic turn amyloid-β is associated with endoplasmic reticulum stress in Alzheimer's disease. Curr. Alzheimer Res. *10*, 11–20.
30. Hoozemans, J.J., Veerhuis, R., Van Haastert, E.S., Rozemuller, J.M., Baas, F., Eikelenboom, P., and Scheper, W. (2005). The unfolded protein response is activated in Alzheimer's disease. Acta Neuropathol. *110*, 165–172.
31. Kondo, T., Asai, M., Tsukita, K., Kutoku, Y., Ohsawa, Y., Sunada, Y., Imamura, K., Egawa, N., Yahata, N., Okita, K., et al. (2013). Modeling Alzheimer's disease with iPSCs reveals stress phenotypes associated with intracellular Aβ and differential drug responsiveness. Cell Stem Cell *12*, 487–496.
32. Lee, S.J., Lee, I., Lee, J., Park, C., and Kang, W.K. (2014). Statins, 3-hydroxy-3-methylglutaryl coenzyme A reductase inhibitors, potentiate the anti-angiogenic effects of bevacizumab by suppressing angiopoietin2, BiP, and Hsp90α in human colorectal cancer. Br. J. Cancer *111*, 497–505.
33. Gorbatyuk, M.S., Shabashvili, A., Chen, W., Meyers, C., Sullivan, L.F., Salganik, M., Lin, J.H., Lewin, A.S., Muzyczka, N., and Gorbatyuk, O.S. (2012). Glucose regulated protein 78 diminishes alpha-synuclein neurotoxicity in a rat model of Parkinson disease. Mol. Ther. *20*, 1327–1337.
34. Salganik, M., Sergeyev, V.G., Shinde, V., Meyers, C.A., Gorbatyuk, M.S., Lin, J.H., Zolotukhin, S., and Gorbatyuk, O.S. (2015). The loss of glucose-regulated protein 78 (GRP78) during normal aging or from siRNA knockdown augments human alpha-synuclein (α-syn) toxicity to rat nigral neurons. Neurobiol. Aging *36*, 2213–2223.
35. Little, E., Ramakrishnan, M., Roy, B., Gazit, G., and Lee, A.S. (1994). The glucose-regulated proteins (GRP78 and GRP94): functions, gene regulation, and applications. Crit. Rev. Eukaryot. Gene Expr. *4*, 1–18.
36. Li, Y., Xu, S., Zhang, Q., Li, L., Lai, L., Zheng, T., Su, J., Yang, N., and Li, Y. (2014). Cytotoxicity study on SH-SY5Y cells cultured at high glucose levels and treated with bupivacaine. Mol. Med. Rep. *9*, 515–520.
37. Claxton, A., Baker, L.D., Wilkinson, C.W., Trittschuh, E.H., Chapman, D., Watson, G.S., Cholerton, B., Plymate, S.R., Arbuckle, M., and Craft, S. (2013). Sex and ApoE genotype differences in treatment response to two doses of intranasal insulin in adults with mild cognitive impairment or Alzheimer's disease. J. Alzheimers Dis. *35*, 789–797.
38. Bassil, F., Fernagut, P.O., Bezard, E., and Meissner, W.G. (2014). Insulin, IGF-1 and GLP-1 signaling in neurodegenerative disorders: targets for disease modification? Prog. Neurobiol. *118*, 1–18.
39. Uemura, E., and Greenlee, H.W. (2006). Insulin regulates neuronal glucose uptake by promoting translocation of glucose transporter GLUT3. Exp. Neurol. *198*, 48–53.
40. Biessels, G.J., and Kappelle, L.J.; Utrecht Diabetic Encephalopathy Study Group (2005). Increased risk of Alzheimer's disease in Type II diabetes: insulin resistance of the brain or insulin-induced amyloid pathology? Biochem. Soc. Trans. *33*, 1041–1044.
41. Slack, C., Foley, A., and Partridge, L. (2012). Activation of AMPK by the putative dietary restriction mimetic metformin is insufficient to extend lifespan in *Drosophila*. PLoS ONE *7*, e47699.
42. Jagannathan, S., Abdel-Malek, M.A., Malek, E., Vad, N., Latif, T., Anderson, K.C., and Driscoll, J.J. (2015). Pharmacologic screens reveal metformin that suppresses GRP78-dependent autophagy to enhance the anti-myeloma effect of bortezomib. Leukemia *29*, 2184–2191.
43. Hsu, C.C., Wahlqvist, M.L., Lee, M.S., and Tsai, H.N. (2011). Incidence of dementia is increased in type 2 diabetes and reduced by the use of sulfonylureas and metformin. J. Alzheimers Dis. *24*, 485–493.
44. Moore, E.M., Mander, A.G., Ames, D., Kotowicz, M.A., Carne, R.P., Brodaty, H., Woodward, M., Boundy, K., Ellis, K.A., Bush, A.I., et al.; AIBL Investigators (2013). Increased risk of cognitive impairment in patients with diabetes is associated with metformin. Diabetes Care *36*, 2981–2987.
45. Picone, P., Nuzzo, D., Caruana, L., Messina, E., Barera, A., Vasto, S., and Di Carlo, M. (2015). Metformin increases APP expression and processing via oxidative stress, mitochondrial dysfunction and NF-κB activation: Use of insulin to attenuate metformin's effect. Biochim. Biophys. Acta *1853*, 1046–1059.
46. Osterwalder, T., Yoon, K.S., White, B.H., and Keshishian, H. (2001). A conditional tissue-specific transgene expression system using inducible GAL4. Proc. Natl. Acad. Sci. USA *98*, 12596–12601.
47. Kinghorn, K.J., Castillo-Quan, J.I., Bartolome, F., Angelova, P.R., Li, L., Pope, S., Cochemé, H.M., Khan, S., Asghari, S., Bhatia, K.P., et al. (2015). Loss of PLA2G6 leads to elevated mitochondrial lipid peroxidation and mitochondrial dysfunction. Brain *138*, 1801–1816.
48. Simonsen, A., Cumming, R.C., Brech, A., Isakson, P., Schubert, D.R., and Finley, K.D. (2008). Promoting basal levels of autophagy in the nervous system enhances longevity and oxidant resistance in adult *Drosophila*. Autophagy *4*, 176–184.
49. Veleri, S., Rieger, D., Helfrich-Förster, C., and Stanewsky, R. (2007). Hofbauer-Buchner eyelet affects circadian photosensitivity and coordinates TIM and PER expression in *Drosophila* clock neurons. J. Biol. Rhythms *22*, 29–42.
50. Levine, J.D., Funes, P., Dowse, H.B., and Hall, J.C. (2002). Signal analysis of behavioral and molecular cycles. BMC Neurosci. *3*, 1.
51. Gilestro, G.F., and Cirelli, C. (2009). pySolo: a complete suite for sleep analysis in *Drosophila*. Bioinformatics *25*, 1466–1467.

Current Biology, Volume *26*

Supplemental Information

Increased Glucose Transport into Neurons Rescues Aβ Toxicity in *Drosophila*

Teresa Niccoli, Melissa Cabecinha, Anna Tillmann, Fiona Kerr, Chi T. Wong, Dalia Cardenes, Alec J. Vincent, Lucia Bettedi, Li Li, Sebastian Grönke, Jacqueline Dols, and Linda Partridge

Supplemental Figures

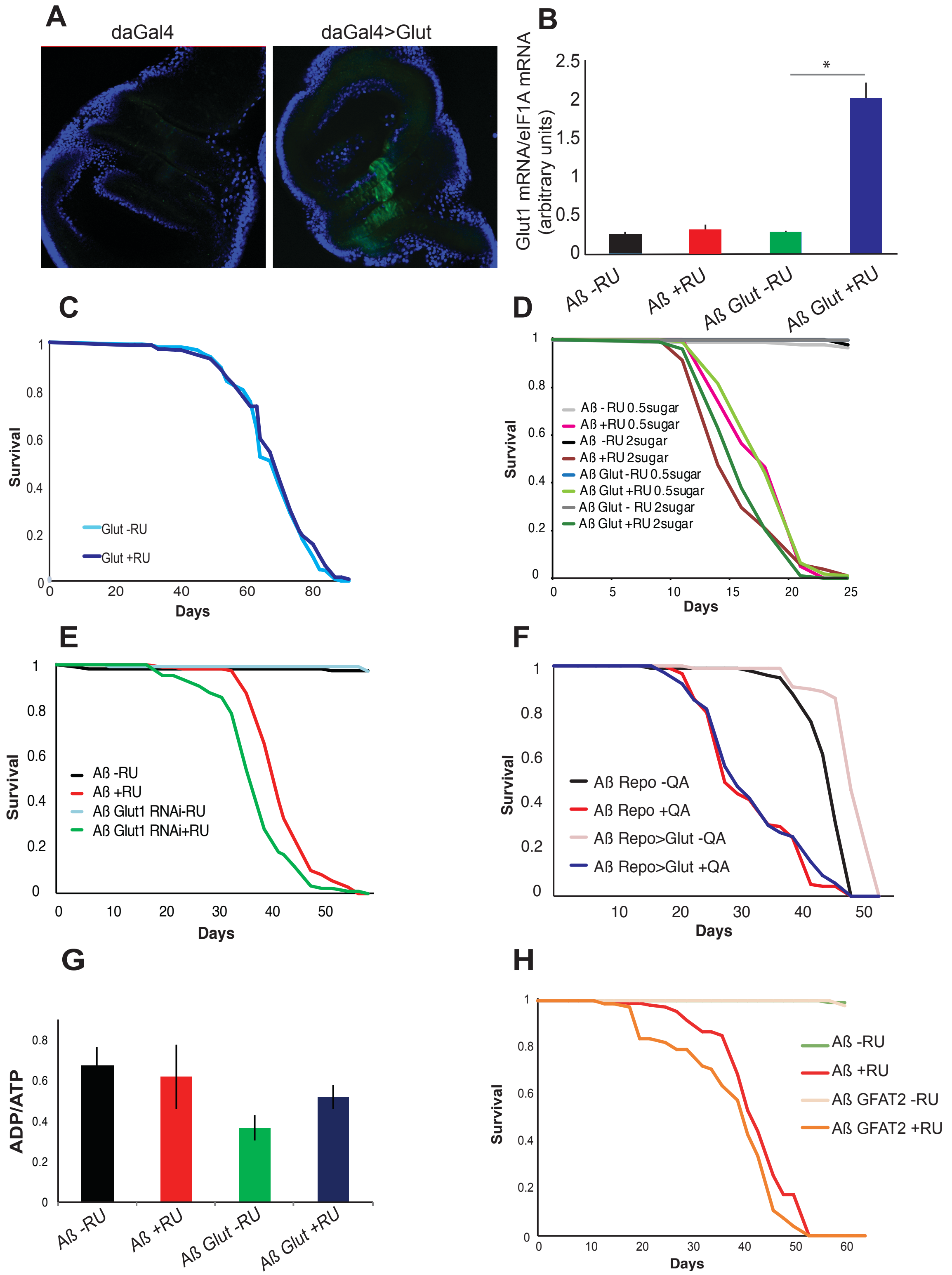

Supplemental Figure 1, related to Fig 1. A. Uptake of the fluorescent glucose analogue 2-NBDG (green) in imaginal discs expressing Glut1 and driver alone control (nuclei marked with DAPI in blue). **B**. Glut1 mRNA levels measured by qPCR (relative to eIF1A), showing increase in Glut1 levels in response to RU in the UAS-Glut1 line. Data are presented as means ±SEM (n=3) (* $p \leq 0.01$ by ANOVA) Genotypes: *UAS Aβ; elavGS, UAS Aβ UAS Glut1; elavGS*. **C**. Survival curve of flies expressing Glut1 (+RU) and uninduced control (-RU) in adult neurons.

Genotype: *UAS-Glut1; elavGS.* **D.** Survival curve of flies expressing Aß or Aß Glut1 in adult neurons (+RU) and their uninduced control (-RU), at 29C, on different sucrose concentrations (2=10% and 0.5=2.5%). Sugar concentration differentially modifies the response to RU in Aß Glut1 flies relative to the Aß alone expressing flies ($p<0.05$ by Cox proportional hazards for the interaction between RU, Sugar and genotype). For the individual sugar concentrations comparison on +RU, the lifespans on 0.5x were not statistically significantly different, whereas for 2x sugar, the AßGlut1 lifespan was statistically significantly longer than the Aß alone lifespan ($p\leq0.01$ by Cox proportional hazards analysis). **E.** Survival curves of Aß expressing flies in the presence and absence of Glut1. Aß Glut1RNAi is significantly shorter lived than the Aß alone ($p<0.001$ by log-rank for difference between Aß and Aß Glut1RNAi +RU). Genotypes*: wv; UAS Aß; elavGS, wv; UAS Aß; UAS Glut1RNAi/ elavGS.* **F.** Survival curves of flies expressing Glut1 in glia under the control of the repo promoter, while Aß was induced by feeding flies QA. Genotypes*: QUAS Aß; repoGal4 nSybQF2 tubQS, QUAS Aß/UAS Glut1; repoGal4 nSybQF2 tubQS* **G.** ADP/ATP ratio in day 19 fly heads expressing Aß or Aß Glut1.There is no statistical difference between the samples. Data are presented as means ±SEM (n=4). Genotypes: *UAS Aß; elavGS, UAS Aß/UAS Glut1; elavGS*. **H**. GFAT2 expression shortens Aß lifespan. Survival curves of flies expressing Aß or Aß GFAT2 in neurons induced after eclosion (+RU) and uninduced controls (-RU). Genotypes: *UAS Aß/ UAS GFAT2; elavGS, UAS Aß; elavGS*. ($p<0.001$ for difference between Aß +RU and AßGFAT2 +RU by log-rank test)

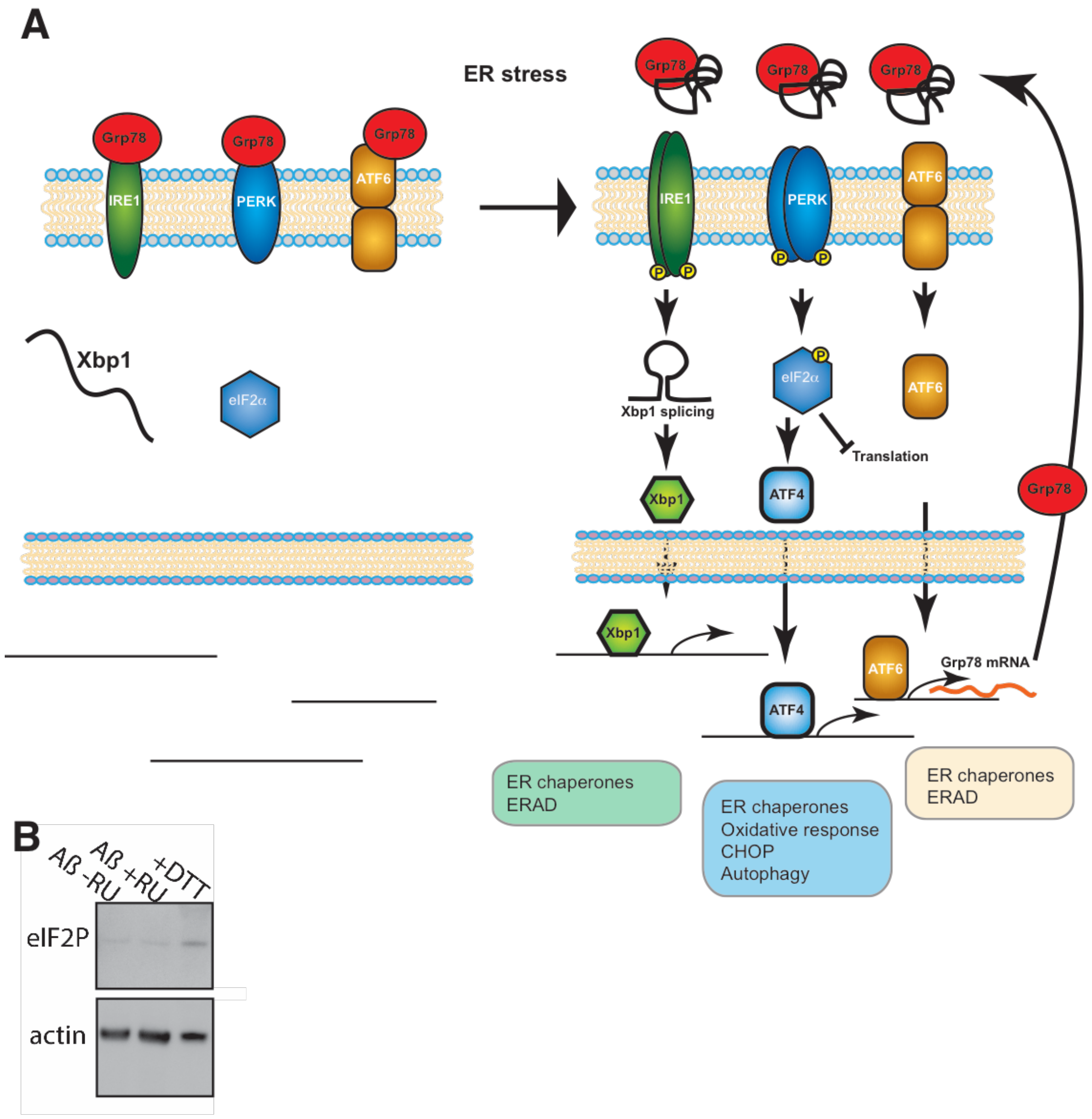

Supplemental Figure 2, related to Fig 2. A. Schematic representation of the UPR. The UPR is mediated by 3 trans-membrane signalling components: inositol-requiring enzyme 1 (IRE1), activating transcription factor 6 (ATF6) and protein kinase RNA-like ER kinase (PERK). Under normal physiological conditions, these are bound and inhibited by Grp78/BiP. During ER stress mis-folded proteins titrate away Grp78 thus activating the signalling cascades downstream of IRE1, ATF6 and PERK. This leads to phosphorylation of eIF2a by PERK and global attenuation of protein translation and induction of a set of downstream transcription factors which lead to the production of proteins involved in re-instating ER proteostasis. IRE1 induces Xbp1 splicing, leading to the translation of an active Xbp1transcription factor, ATF6 is cleaved in the Golgi to give a cytosolic ATF6 fragment, which also enters the nucleus to induce transcription, and PERK induces the expression of ATF4. All these induce the expression of chaperones, factors involved in ERAD (ER-associated protein degradation), autophagy, redox and, eventually, apoptosis. **B.**Western blot of head samples from flies expressing Aß (+RU) and their uninduced controls (-RU) as well as heads treated with 5mM DTT in Schneider's medium for 4 hours, to induce the UPR. Genotypes: *UAS Aß; elavGS.*.

Supplemental Experimental Procedures

Generation of new transgenic lines

The Glut1 open reading frame from clone LD20062 (DSHB, Iowa) was digested with EcoR1 and Xho1 restriction enzymes and cloned into pUAST. The pUAST-Glut1 construct was used to generate germline transgenics in the w1118 background (Fly Facility, Department of Genetics, University of Cambridge). A number of transgenics were generated and one line carrying UAS-Glut1 (line1) on the second chromosome was used for this study. Another line UAS-Glut1 (line8) was used to confirm the initial phenotype (data not shown).

The GFAT2 open reading frame was amplified from genomic DNA using primers CTACTCCACGGTCACCGACTTG+ CACCATGTGTGGCATATTCGCGT and topo cloned into pENTR/ TOPO entry vector (Invitrogen K2400-20) following the manufacturer's protocol and from this transferred to vector pUASTattB with an LR clonase (Invitrogen 11791-043) according to the manufacturer's protocols. To generate transgenic flies, the vector was integrated into the attP40 landing site using φC31 and attP/attB targeted integration system.

The QUAS-Aß42 Arctic flies were generated by amplifying the Aß42 Arctic sequence from plasmid pMT AßArc (a generous gift from Damian Crowther) with primers CACCATGGCGAGCAAAGTCTC and TTACGCAATCACCACGCCGC and cloned into vector pQUAS. pQUAS-Aß42 Arctic was used to generate germline transgenics in the w1118 background (Fly Facility, Department of Genetics, University of Cambridge). A number of transgenics were generated and one line carrying QUAS-Aß42 Arc (QUAS-Aß) on the second chromosome was used for this study (line D).

Glucose uptake assay

Glucose uptake was carried out based on [S1]. Briefly, imaginal discs were dissected from L3 larvae and incubated in PBS with 2.5mg/ml 2-NBDG (Molecular Probes N13195) for 20 minutes, then washed twice in ice cold PBS, mounted in Vectashield containing DAPI and imaged immediately on a Zeiss LSM510 inverted confocal microscope.

ADP/ATP

20 fly heads were homogenised in 30µl of TCA with glass beads in a ribolyser. Samples were spun at 11,500g for 15 minutes at 4ºC. Supernatants were neutralised by diluting 1:10 in 1M Tris HCl pH 8. 50µl of sample was used to measure ADP and ATP with Abcam's ADP/ATP Ratio Assay Kit (ab65313), according to the manufacturer's protocol. Samples were loaded in duplicate, and 4 biological repeats were used for each genotype. Values are expressed as the mean of those replicates +/-SEM, samples were compared with ANOVA.

Quantitative PCR

Total RNA was extracted from 20-25 fly heads per sample using TRIzol® (GIBCO) according to the manufacturer's instructions. The concentration of total RNA purified for each sample was measured using an Eppendorf biophotometer. One microgram of total RNA was then subjected to DNA digestion using DNase I (Ambion), immediately followed by reverse transcription using the SuperScript® II system (Invitrogen) with oligo(dT) primers. Quantitative PCR was performed using the PRISM 7000 sequence-detection system (Applied Biosystems), SYBR® Green (Molecular Probes), ROX Reference Dye (Invitrogen), and HotStarTaq (Qiagen) by following the manufacturer's instructions. Each sample was analysed in duplicate and values are the mean of three-four independent biological repeats +/- SEM. Primers used were:

Glut1 TTACCGCGGAGCTCTTCTCC +GCCATCCAGTTGACCAGCAC
Aß42 CGATCCTTCTCCTGCTAACC+ CACCATCAAGCCAATAATCG
Grp78 TCTTGTACACACCAACGCAGG+ CAAGGAGCTGGGCACAGTGA
eIF1A ATCAGCTCCGAGGATGACGC + GCCGAGACAGACGTTCCAGA
Spliced Xbp1 CCGAACTGAAGCAGCAACAGC + GTATACCCTGCGGCAGATCC [S2]

Supplemental References

S1. Zou, C., Wang, Y., and Shen, Z. (2005). 2-NBDG as a fluorescent indicator for direct glucose uptake measurement. Journal of biochemical and biophysical methods *64*, 207-215.

S2. Maor, G., Rencus-Lazar, S., Filocamo, M., Steller, H., Segal, D., and Horowitz, M. (2013). Unfolded protein response in Gaucher disease: from human to Drosophila. Orphanet J Rare Dis *8*, 140.
